# Promotion of couples' voluntary counselling and testing for HIV through influential networks in two African capital cities

**DOI:** 10.1186/1471-2458-7-349

**Published:** 2007-12-11

**Authors:** Susan Allen, Etienne Karita, Elwyn Chomba, David L Roth, Joseph Telfair, Isaac Zulu, Leslie Clark, Nzali Kancheya, Martha Conkling, Rob Stephenson, Brigitte Bekan, Katherine Kimbrell, Steven Dunham, Faith Henderson, Moses Sinkala, Michel Carael, Alan Haworth

**Affiliations:** 1 The Rwanda-Zambia HIV Research Group, 1520 Clifton Road, Emory University, Atlanta, GA 30322, USA; 2 Hubert Department of Global Health, Rollins School of Public Health, Emory University, 1518 Clifton Road, Atlanta, GA 30322, USA; 3 University Teaching Hospital, Lusaka, Zambia; 4 Department of Biostatistics, School of Public Health, University of Alabama at Birmingham, 1530 3^rd ^Ave S., Birmingham, AL 35294-0022, USA; 5 Department of Public Health Education, University of North Carolina at Greensboro, 437 HHP Building, Greensboro, NC 27402-6170, USA; 6 Department of Pediatrics, School of Medicine, University of Southern California, 4650 Sunset Blvd., CHLA MS 30, Los Angeles, CA 90027, USA; 7 School of Medicine, Harvard University, 25 Shattuck Street, Boston, MA 02115, USA; 8 Lusaka Urban District Health Management Team, Makishi Road, PO Box 50827, Lusaka, Zambia; 9 Monitoring and Evaluation Unit, UNAIDS, 20 Avenue Appia, CH-1211 Geneva 27, Switzerland; 10 Counseling Services Unit, Ministry of Health, PO Box 30205, Lusaka, Zambia

## Abstract

**Background:**

Most new HIV infections in Africa are acquired from cohabiting heterosexual partners. Couples' Voluntary Counselling and Testing (CVCT) is an effective prevention strategy for this group. We present our experience with a community-based program for the promotion of CVCT in Kigali, Rwanda and Lusaka, Zambia.

**Methods:**

Influence Network Agents (INAs) from the health, religious, non-governmental, and private sectors were trained to invite couples for CVCT. Predictors of successful promotion were identified using a multi-level hierarchical analysis.

**Results:**

In 4 months, 9,900 invitations were distributed by 61 INAs, with 1,411 (14.3%) couples requesting CVCT. INAs in Rwanda distributed fewer invitations (2,680 vs. 7,220) and had higher response rates (26.9% vs. 9.6%), than INAs in Zambia. Context of the invitation event, including a discreet location such as the INA's home (OR 3.3–3.4), delivery of the invitation to both partners in the couple (OR 1.6–1.7) or to someone known to the INA (OR 1.7–1.8), and use of public endorsement (OR 1.7–1.8) were stronger predictors of success than INA or couple-level characteristics.

**Conclusion:**

Predictors of successful CVCT promotion included strategies that can be easily implemented in Africa. As new resources become available for Africans with HIV, CVCT should be broadly implemented as a point of entry for prevention, care and support.

## Background

In 2006 more than 65% of the 4.3 million new cases of HIV occurred in Sub-Saharan Africa [1]. In this region, most transmission is heterosexual, occurring predominately between cohabiting partners [2]. Among pregnant women tested with their partners in antenatal clinics in Lusaka, the capital of Zambia, 19% of couples had two HIV+ partners ("concordant +") and 17% had one HIV+ and one HIV- partner ("discordant"). In Kigali, the capital of Rwanda, 8% of couples were concordant + and 9% were discordant [3]. These results are similar to those reported in couples' voluntary HIV counselling and testing (CVCT) centres [4-7], cluster sampling surveys [8], and to those reported for couples in neighbouring Kenya and Tanzania [9].

Cohabiting couples in Africa now represent the world's largest HIV risk group [10,11]. VCT is a cost-effective method of reducing HIV transmission [12-14] and traditional clinic-based services have recently expanded to include home based, workplace and mobile counselling strategies [13,15-20]. Though VCT is particularly effective when partners are tested together, joint testing remains rare in Africa. Promotional strategies, including door-to-door outreach by community workers and weekend CVCT services in antenatal clinics, have been shown to increase the number of couples tested [3-5,15]. While these efforts show that CVCT can be de-centralized and implemented in a variety of settings, they have not proven to be sustainable after research funding has ended.

Several factors affect demand for and supply of CVCT. Demand for CVCT is low because of the belief that monogamy is 'safe', fear of stigma, gender inequality, and lack of knowledge of the availability of CVCT [4,6,21-23]. Given the low demand, policymakers and other influential groups have not promoted CVCT. In turn, funding agencies have not supported CVCT services, further compromising supply and ensuring low utilization. Given what we know about the beneficial impact of CVCT [24], it is critical that this continuing cycle of low demand and low supply be interrupted [11].

The importance of social networks and community leader involvement in changing attitudes towards HIV/AIDS has been shown to have significant impact on risk perception among individuals in Southern Africa [25-27]. We present here the results of an intervention using influential people to overcome ignorance and stigma, establish the importance of being tested as couples, and invite couples to attend CVCT services in two African capital cities, Kigali, Rwanda and Lusaka, Zambia.

## Methods

The study was initiated by the Rwanda Zambia HIV Research Group (RZHRG), in collaboration with the Ministry of Health Treatment and Research AIDS Centre (TRAC) in Rwanda and the University Teaching Hospital (UTH) in Zambia and approved by Institutional Review Boards in Rwanda, Zambia, and the US. The components of the intervention included recruitment and training of Influence Network Agents (INAs) to promote CVCT, and provision of CVCT services at stand-alone RZHRG facilities. The study took place from January to July, 2003.

### Recruitment and training of INAs

RZHRG staff and collaborators at UTH and TRAC identified and referred candidate INAs considered influential in the communities near the CVCT centres. A four-day training began with orientation including observation of the CVCT process and a demonstration of door-to-door promotion by project community workers (CWs). Given that INAs might be reluctant to be tested by colleagues, testing for HIV was offered but not required as part of training. Standard operating procedures (SOPs) were developed to guide all INA related training and outreach activities. INAs then completed a demographic questionnaire, participated in focus groups with 4–8 other INAs, and attended formal didactic training on HIV, CVCT, outreach skills, and the logistics of invitation distribution and data collection. Each INA was asked to choose the category that best described their role when distributing invitations: health care, religious, non-governmental/community-based organization (NGO/CBO), or private sector. While the recruitment goal was to have roughly equal numbers of INAs in each of the four categories, individual INAs may have identified more strongly with a role other than the one they were recruited in. For example a business owner may have also been a church elder and may have felt that the latter role would be more important when promoting CVCT.

### INA data collection

Invitations distributed to couples described the location of the centre and CVCT procedures. Each invitation was identified by a numeric code including a unique INA identifier and an invitation number. A corresponding "invitation receipt" with the same numeric code allowed the INA to record basic demographic data about the invited couples, including age of man and woman, duration of cohabitation, number of children, and residential neighbourhood. No names or couple identifiers were recorded. INAs also recorded date and time of the invitation; location (couple home, INA home, workplace, community); the relationship of the INA to the invitee (family member, friend, professional or social contact, or just met/unknown); who received the invitation (couple, man, woman); and whether the invitation was preceded by a public endorsement for CVCT by the INA or another community leader.

### INA follow-up

Weekly follow-up meetings were held to discuss challenges INAs encountered during their work. Completed "invitation receipts" were collected. INAs were encouraged to begin slowly (10 invitations/week) and build up to a manageable volume. The maximum number of invitations distributed overall was determined by the capacity of the testing centre. A process oriented method of payment was employed. INAs received a small incentive payment for each invitation delivered, a larger payment for any couples who attended a CVCT session and transportation costs to facilitate attendance at the weekly meetings.

### CVCT procedures

CVCT procedures have been previously described [4]. Briefly, each morning 10–25 couples participated in a group discussion led by a counsellor. This was followed by joint confidential pre-test counselling sessions, informed consent procedures if HIV testing was desired, phlebotomy, and lunch while rapid HIV testing was performed. These services were provided free of charge and included a reimbursement of transportation expenses and child care. Results and joint post-test counselling were provided in the afternoons. All blood tubes and result forms were coded with a couple testing ID number. CVCT clients were a mixture of INA-invited and 'walk-in' couples; the data presented here are limited to couples invited by INAs. Invitations were collected from INA-invited couples at the time of pre-test counselling, and the invitation number was linked to the couple testing ID number for the purposes of this analysis.

### Statistical Analysis

Data were entered in Access databases and analyzed with the SAS statistical package (version 9.0; Statistical Analysis Software, North Carolina, USA).

Two outcomes are examined to measure the 'success' of INA invitations. First, we examined predictors of the number of invitations distributed per INA using standard linear regression models. Second, we tracked which couples came for testing and examined the predictors of a 'successful' invitation. Because invitations are nested or clustered within individual INAs and therefore not independent observations, a hierarchical or nested random-effects model was used to estimate error variability at both the INA and couple levels simultaneously. We used the Hierarchical Linear Modelling (HLM) analysis system developed by Raudenbush and colleagues [28] to accomplish this objective. Each invitation was coded as successful or unsuccessful based on whether the couple later attended the CVCT centre. This binary outcome variable was analyzed as a function of invitation-level and INA-level predictors. The logit link function was applied to this binary outcome variable, making the HLM analysis analogous to a hierarchical or clustered extension of standard logistic regression analysis. The iterative, penalized quasi-likelihood (PQL) estimation method was used, and the unit-specific estimates, odds ratios, confidence intervals, and t-tests were used to evaluate each predictor. Variables to be considered in the multivariate analysis were selected based on the results of univariate analysis, using p < .05 as the criteria.

## Results

### INA characteristics

In Kigali, 28 INAs included 10 men and 18 women, with 5 religious group affiliates, 8 health-care providers, 5 NGO/CBO members, and 10 private sector representatives. In Lusaka, 33 INAs included 20 men and 13 women, of whom 10 were affiliated with religious groups, 8 were health-care providers, 6 worked with NGO/CBO, and 9 were from the private sector. The mean age of INAs was 40 years in both cities (range 20–65) and they lived in the catchment area of the CVCT centres. Forty-one of the 61 INAs (67%) received HIV counselling and testing prior to or during the training period, of whom 26 were tested with their spouses.

### Invitations to attend CVCT

Invitations were distributed to 9,900 couples (2,680 in Rwanda, 7,220 in Zambia) in a 4 month period. The average ages of invited men and women in each country were similar (Rwanda: Men = 35.6 years, Women = 29.6 years; Zambia: Men = 34.5 years, Women = 28.1 years). Couples had been married or cohabiting for a mean of 8.0 years (Rwanda = 7.8 years, Zambia = 8.0 years), and had an average of 2.5 children (Rwanda = 2.5, Zambia = 2.4).

Table 1 presents the contextual characteristics of CVCT invitations given by INAs, stratified by category and city. Invitations in Rwanda and Zambia were similar in that 56% were given to someone known to the INA, including professional or social contacts, friends, or family members. Rwandan INAs were more likely than Zambian counterparts to invite both members of the couple together (33% *vs*. 25%) rather than the man or woman alone. In both cities, 60% of invitations were delivered at the client's home, with the remainder given at the INA home, workplace or clinic, or in the community (including church, market, social gathering, or other). Zambian INAs were more likely to deliver invitations accompanied by their spouse (12% of invitations) or another INA (8%), while Rwandan INAs tended to work alone (95% of invitations). Invitations in Rwanda had more often been preceded by a public endorsement of CVCT than those in Zambia (11% *vs*. 6%).

**Table 1:** Contextual characteristics of CVCT invitations given by INAs in four categories in Rwanda and Zambia

	**Rwanda**					**Zambia**				
		
	**CBO/NGO**	**Religious**	**Health**	**Private**	**Total**	**CBO/NGO**	**Religious**	**Health**	**Private**	**Total**
			
**N INAs**	5	5	8	10	28	6	10	8	9	33
**N invitations**	472	174	944	1090	2680	1337	2184	1757	1942	7220
**Relationship to INA**	%	%	%	%	%	%	%	%	%	%
Family	1	5	1	2	2	7	4	5	5	5
Friends	11	10	20	18	17	14	5	15	7	10
Professional	10	30	9	24	16	22	9	36	24	22
Social	24	24	19	20	21	12	31	21	7	19
Just met	44	25	50	30	39	45	50	23	56	44
Other	11	7	1	6	5	0	1	0	1	1

**Person(s) invited**										
Couple	29	25	40	31	33	23	28	19	29	25
Man	36	26	32	39	35	44	26	37	43	37
Woman	35	49	29	30	32	33	47	44	28	39

Place of invitation**										
Couple home	42	30	59	72	60	51	81	40	61	60
INA home	26	18	4	5	9	11	6	3	4	6
Community	19	18	19	9	15	23	8	12	20	15
Work place	13	34	18	15	17	14	5	46	16	19

**Person inviting**										
INA	100	100	98	91	95	74	79	93	76	81
+Spouse	0	0	0.5	8.5	4	14	7	3	23	12
+INA	0	0	1.5	0.5	1	12	14	4	1	8

**Public endorsement**										
Yes	5	24	19	5	11	7	2	14	2	6
No	95	76	80	95	89	93	98	86	98	94

*Rounding results in some totals of 99% or 101%

**Community = Church, Market, Social Gathering, and Other

The relationships between invitees and INAs varied across categories in the two cities. In Rwanda, religious and private sector INAs were more likely to invite people they knew (68% and 64%, Table 1) than CBO/NGO and health sector (45% and 49%) INAs, while religious and private sector Zambian INAs distributed half or more of their invitations to people they had just met (50% and 56%). Invitations from Rwandan health care INAs were the most likely to be given to couples (40%) compared with those from other INAs in Rwanda (25–31%), while religious and private sector INAs were more likely to target couples in Zambia (28%–29%) than their NGO/CBO (23%) or health sector (19%) counterparts. Compared to other categories, NGO/CBO INAs delivered a comparatively larger proportion of their invitations in their own homes (26% in Rwanda, 11% in Zambia), as did religious INAs in Rwanda (18%). Fewer than 6% of invitations delivered by the other INA country-categories were delivered in the INA's home. Workplace invitations were favoured by health care INAs in Zambia (46%) and religious INAs in Rwanda (34%), and these two groups were also more likely than their in-country counterparts to have invitations preceded by a public endorsement (14% and 24%, respectively).

### Response to invitations

Of 9,900 couples who received invitations, 1,411 (14.3%) requested CVCT. The response rates, or percent of invited couples who came for testing, were much higher in Rwanda, where 721 of the 2,680 (26.9%) invited couples came in to be tested, than in Zambia, where only 690 of the 7,220 (9.6%) couples were tested. These rates were significantly different (t (9,839) = 6.55, p < .0001). Table 2 details the number of invitations distributed, the number of invited couples who received CVCT, and the response rate for each INA, grouped by country and category and ordered by decreasing number of couples tested. Zambian INAs distributed more invitations (mean 219, S.D. 93) than Rwandan INAs (mean 96, S.D. 99), with greater variation among Rwandan INAs. Multiple regression analyses within each country separately revealed that the number of invitations distributed did not vary significantly by INA category, gender, age, marital status, or whether the INA had received HIV testing.

**Table 2:** Number of invitations issued and couples tested by INA and category for Rwanda and Zambia

		**Rwanda**				**Zambia**			
			
**Category**		**INA**	**Invitations Issued**	**Couples Tested**	**%**	**INA**	**Invitations Issued**	**Couples Tested**	**%**
	
**Health**		**N = 8**				**N = 8**			
		1	233	66	28	1	184	30	16
		2	288	57	20	2	223	22	10
		3	263	20	8	3	244	18	7
		4	112	20	18	4	236	18	8
		5	19	19	100	5	242	16	7
		6	14	6	43	6	228	12	5
		7	4	3	75	7	247	7	3
		8	11	0	0	8	153	4	3
			
	**Subtotal**		944	191	20		1757	127	7
	
**Private Sector**		**N = 10**				**N = 9**			
		1	208	94	45	1	243	34	14
		2	338	68	20	2	247	26	11
		3	116	43	37	3	217	25	12
		4	205	26	13	4	228	21	9
		5	42	21	50	5	159	13	8
		6	64	19	30	6	212	11	5
		7	19	12	63	7	214	8	4
		8	26	10	38	8	174	5	3
		9	66	7	11	9	248	2	1
		10	6	4	67	-	-	-	-
			
	**Subtotal**		1090	304	28		1942	145	7
	
**NGO/CBO**		**N = 5**				**N = 6**			
		1	198	90	45	1	247	66	27
		2	148	30	20	2	249	34	14
		3	67	16	24	3	179	32	18
		4	48	14	29	4	214	28	13
		5	11	6	55	5	211	15	7
		-				6	237	10	4
			
	**Subtotal**		472	156	33		1337	185	14
	
**Religious**		**N = 5**				**N = 10**			
		1	48	33	69	1	208	77	37
		2	64	24	38	2	202	39	19
		3	10	8	80	3	237	36	15
		4	48	3	6	4	268	24	9
		5	4	2	50	5	245	20	8
		-	-	-	-	6	163	12	7
		-	-	-	-	7	219	10	5
		-	-	-	-	8	241	9	4
		-	-	-	-	9	208	4	2
		-	-	-	-	10	193	2	1
			
	**Subtotal**		174	70	40		2184	233	11
**TOTAL**			**2680**	**721**	**27**		**7220**	**690**	**10**

Although Rwandan INAs distributed fewer invitations, they had a higher response rate. As a result, the average number of couples tested per INA was not significantly different in the two countries (26 in Rwanda, 21 in Zambia). The main determinant of INA performance in Rwanda was the number of invitations distributed, as response rates were > = 20% for 22/28 INAs. In Zambia, only 2/33 INAs had response rates > = 20% and only 12/33 exceeded response rates of 10%. In Zambia, all INAs distributed a large number of invitations but had fewer invitees present for CVCT services. In both countries, there were a few 'high performing' individuals, who had both high numbers of invitations and a high response rate.

### Predictors of successful invitations

Table 3 summarizes the effects of univariate HLM analyses predicting response rates in each country separately. For categorical variables (e.g., location of invitation, INA category), dummy-coded vectors were entered as a set with the last category serving as a reference for the other categories. None of the INA-level measures were predictive of successful invitations in either country.

**Table 3:** Summary of univariate effects from HLM analyses for predicting CVCT response rates in Rwanda and Zambia

	**Rwanda**			**Zambia**		
		
	OR	95% CI	p	OR	95% CI	p
		
**Couple/Invitation Level Predictors**						
Years cohabiting	1.011	0.996 – 1.026	0.141	1.03	1.017 – 1.043	< .001
Number of children	0.991	0.941 – 1.043	0.725	1.005	0.958 – 1.055	0.83
Age of man	1.01	1.009 – 1.011	0.057	1.017	1.008 – 1.026	< .001
Age of woman	1.008	0.996 – 1.021	0.183	1.017	1.007 – 1.027	0.002
						
Place of invitation*						
Couple Home *vs*. Community	1.318	0.944 – 1.839	0.104	1.432	1.047 – 1.958	0.025
INA Home *vs*. Community	3.546	2.281 – 5.524	< .001	3.248	2.212 – 4.769	< .001
Workplace *vs*. Community	1.792	1.225 – 2.621	0.003	1.583	1.104 – 2.269	0.013
						
**Person(s) invited**						
Couple *vs*. Woman	1.559	1.215 – 1.999	0.001	1.918	1.531 – 2.402	< .001
Man *vs*. Woman	1.324	1.053 – 1.665	0.017	1.218	0.983 – 1.510	0.071
						
**Relationship to INA**						
Family member *vs*. Just met	1.503	0.738 – 3.060	0.262	1.984	1.320 – 2.982	0.001
Friend/Social/Professional *vs*. Just met	1.263	1.015 – 1.573	0.036	1.917	1.495 – 2.458	< .001
						
**Public endorsement**	1.535	1.067 – 2.207	0.021	1.435	0.942 – 2.185	0.092
						
**INA level Predictors**						
Religious *vs *. Private	1.536	0.355 – 6.659	0.551	1.278	0.559 – 2.922	0.549
NGO/CBO *vs *. Private	0.966	0.237 – 4.070	0.961	2.09	0.821 – 5.319	0.117
Health *vs*. Private	0.867	0.247 – 3.048	0.817	1.052	0.438 – 2.520	0.907
Gender (M = 1, F = 0)	2.48	0.912 – 6.742	0.07	0.976	0.512 – 1.861	0.94
Age of INA	1.014	0.966 – 1.050	0.572	0.996	0.967 – 1.027	0.806
Married (Y = 1, N = 0)	1.135	0.403 – 3.198	0.803	1.211	0.535 – 2.742	0.635
INA HIV tested (Y = 1, N = 0)	0.923	0.331 – 2.575	0.874	0.633	0.333 – 1.205	0.157

*Workplace = Clinic and Workplace

Community = Church, Market, Social Gathering, and Other

Among invited couples, older age in men was marginally predictive of seeking testing in Rwanda, while older age in men and women and longer cohabitation were significant predictors in Zambia. In both countries, invitations delivered in the INA home were strongly predictive of a successful outcome, and workplace invitations also had a comparatively good yield. In Zambia, invitations delivered at the couples' home were also more likely to result in couples seeking testing compared to the "community" reference group. Invitations delivered to couples were associated with the greatest likelihood of CVCT, and those given to men were more likely to result in testing than those given to women alone. INAs had more success inviting people they knew, compared with people they had just met. Invitations given by the INA accompanied by their spouse or another INA were not associated with a different response rate than those given by the INA alone (not shown). In both countries, invitations delivered after a public endorsement of CVCT were associated with a higher response rate. This association was not significant for Zambia, but remained of interest with an exploratory p-value < 0.10.

Predictor variables with significant univariate effects (p < .05 in either country) were examined in the context of multi-predictor HLM analysis. Because years cohabiting, number of children, age of the man, and age of the woman were all highly correlated with each other (all r's ≥ 60), only the age of the man was included in order to minimize potential multicollinearity. None of the INA-level predictors achieved statistical significance in the univariate analysis, so these variables were excluded from the multi-predictor models.

Table 4 presents the results of the multi-predictor analysis and confirms that invitations given in the INA home, workplace, and (in Zambia only) the couples' home remained independent predictors of CVCT. Similarly, invitations to couples and public endorsement remained predictive in the multivariate model. Invitations to family and to friends and social or professional contacts were independently predictive of success in Zambia but did not remain significant in Rwanda when controlled for the other variables measured.

**Table 4:** Summary of effects from multi-predictor HLM analyses for predicting CVCT response rates- Rwanda and Zambia

	**Rwanda**			**Zambia**		
		
	OR	95% CI	p	OR	95% CI	p
		
**Couple/Invitation Level Predictors**						
Age of man	1.013	1.006 – 1.024	0.015	1.017	1.007 – 1.025	< .001
						
Location of invitation*						
Couple Home *vs*. Community	1.262	0.902 – 1.764	0.200	1.596	1.135 – 2.243	0.008
INA Home *vs*. Community	3.398	2.150 – 5.368	< .001	3.267	2.174 – 4.909	< .001
Workplace *vs*. Community	1.788	1.209 – 2.644	0.004	1.671	1.143 – 2.443	0.008
						
**Person(s) invited**						
Couple *vs*. Woman	1.638	1.253 – 2.142	0.001	1.733	1.363 – 2.202	< .001
Man *vs*. Woman	1.213	0.940 – 1.566	0.137	1.059	0.841 – 1.334	0.623
						
**Relationship to INA**						
Family member *vs*. Just met	1.760	0.844 – 3.672	0.131	1.698	1.110 – 2.599	0.015
Social acquaint. *vs*. Just met	1.070	0.842 – 1.358	0.581	1.810	1.395 – 2.347	< .001
						
**Public endorsement**	1.756	1.140 – 2.708	0.011	1.705	1.075 – 2.704	0.023

*Workplace = Clinic and Workplace

Community = Church, Market, Social Gathering, and Other

## Discussion

Strategies that attempt to modify behaviour remain the cornerstone of HIV prevention efforts [29]. Couples' VCT can provide life-saving benefits, reduce HIV transmission, sexually transmitted infections, and unintended pregnancies among couples [7,9,30-40]. There is now a growing consensus that CVCT should be widely disseminated, but many cultural and logistical obstacles remain [6]. This study confirms that influential members of the community are willing and able to promote CVCT in Africa. Although there were differences in the impact of CVCT promotion between Kigali, Rwanda and Lusaka, Zambia, the predictors of success were similar.

We recruited and trained a broad range of Influence Network Agents to promote CVCT through one-on-one contacts and written invitations. Our previous work successfully used paid Community Workers (CWs) as full-time promoters of CVCT. When CW outreach was discontinued due to achievement of research enrollment targets, attendance at CVCT sites declined [4,5]. The INA model was implemented in order to increase community awareness of CVCT and ensure sustainability.

INAs self-identified with one of four main categories in this study, but many INAs wore more than one 'hat' and used a variety of networks, including friends, family, professional contacts, and social settings to promote CVCT. Zambian INAs were more likely than their Rwandan counterparts to identify their faith-based 'hat' as their most prominent role in CVCT promotion. This may be due to the central role that religion plays in Zambian culture [41].

INAs were most effective when addressing couples, a strategy that removes the pressure on one spouse to carry the message to the other. Delivering invitations in settings that allowed discreet conversation was important because it allowed fear and stigma to be openly discussed. Fewer than one in ten invitations were delivered in INA homes, but of those one third to one half resulted in couples seeking testing. This may also be indicative of the strength of the relationship between the INA and the couple. Locations such as the couples' home and workplace also proved to be more conducive to successful invitation than public locations such as markets, churches, or social gatherings as noted by others involved in VCT/CVCT research in Zambia, Uganda, and Malawi [15-18]

The response rate among couples invited for CVCT by INAs in Kigali was substantially higher than that in Lusaka. Rwandan INAs were more likely to invite people personally known to them, to invite both members of the couple, and to deliver invitations in their own homes than their Zambian colleagues. Invitations in Rwanda were also more likely to be preceded by a public endorsement of CVCT. Other potential explanations for this difference include possible increased receptivity among Rwandan couples, more selective distribution of invitations on the part of Rwandan INAs based on likelihood of attendance, and/or a combination of greater persuasiveness on the part of Rwandan INAs. Factors unrelated to the study no doubt also played a role. Rwandans have one local language, Kinyarwanda. In comparison, 72 dialects from five major language groups in Zambia are spoken in Lusaka. Kigali is also smaller than Lusaka and transport is easier to organize, which reduced the logistical obstacles to CVCT.

Fear of stigma among married couples is common [6,42,43] and was reported by many INAs in the weekly follow-up meetings as an obstacle to their work. Public endorsement of CVCT was used by some INAs to overcome this fear, an effective strategy that addresses psychosocial barriers to accessing clinic services [44]. However, many INAs were reluctant to make public announcements because they had no experience with public speaking and feared being stigmatized themselves. It should also be noted that since more than half of those people being invited to CVCT were known by the INAs, the identification and training of more INAs over time would be necessary in order to have a continued impact throughout the community.

Although the results of this study are encouraging, the fact that three-quarters of invited couples in Kigali and nine in ten couples in Lusaka did not seek CVCT confirms that obstacles remain. Lack of time and the money needed for transportation have been cited as obstacles to VCT [12,20] and CVCT [4]. It may be that had the INAs been able to provide home based testing or been accompanied by someone trained to do so, the uptake of CVCT would have been higher. Strategies such as mobile units and decentralization of CVCT should be developed to bring services closer to the clients.

## Conclusion

HIV prevention and treatment programs in Africa are receiving an infusion of critical new resources from international donors following exciting developments in antiretroviral therapy and other supportive measures. However, until an effective vaccine and low-cost treatment is available to all who need it, the role of prevention cannot be dismissed. During the rapid expansion of perinatal HIV prevention programs in Africa in the last few years, many thousands of pregnant women were offered VCT and provided with nevirapine. Results from these programs have been discouraging. Researchers and policy makers are advocating for the involvement of men in the treatment process through "PMTCT-Plus" programs that include couple counselling [45] and encourage CVCT before pregnancy in order to safeguard women and improve their outcomes when they do become pregnant [46,47].

Our results demonstrate that the INA model is an effective, sustainable way to promote CVCT in Africa. Influential members of the community are willing and able to inform couples of the benefits of CVCT and invite them to come for services. INAs increase community awareness of CVCT and provide an effective alternative to CWs while educating couples. Predictors of invitation success were similar in the two cities we studied and included strategies that can be easily disseminated in sub-Saharan Africa. Further research is needed on the role of higher level authorities in public endorsements and bringing services to couples in the most desirable form.

Unless systematic efforts are made to address the social, political and cultural obstacles to CVCT, the gap between research and public health policy and practice will not be bridged. Government and community leaders must endorse CVCT, and service providers must acquire skills to counsel couples. Wherever VCT is provided to cohabiting adults, adding a "C" to VCT will save lives.

## Competing interests

The authors declare that they have no competing interests.

## Authors' contributions

SA is the Principal Investigator for the Rwanda Zambia HIV Research Group; EK is the Senior Co-Investigator of Projet San Francisco and EC is the Senior Co-Investigator of the Zambia Emory HIV Research Project. Each participated in all aspects of the study, from study design to creation of instruments and data analysis and reviewed the manuscript. DLR contributed to the design of the study and performed statistical data analysis. JT is an expert in community based research and LC is a behavioral scientist; both contributed to the design of the study, development of the data collection instruments and standard operating procedures, data analysis, and manuscript preparation. IZ and NK oversaw all recruitment efforts in the field and were an active part of the paper revision process while completing their Master's in Public Health degrees in the United States. BB is a physician and public health practitioner who contributed to development of the study design and analysis of the INA level data. KK, medical student, SD, and FH supervised selection and training of INAs, data collection and ongoing monitoring and evaluation analyses during the study. MS, District Director of Health for Lusaka led the dialogues with city leaders in the politico-administrative realm, as well as contacts with community leaders in preparation for the study along with EK. They were responsible for on-site supervision of all activities during the study, and for revision of the manuscript. MCarael is a Sociologist with more than 30 years of experience with the design and implementation of HIV prevention programs in Africa. He participated in development of the grant proposal and study design, and in drafting the manuscript. AH, MConkling and RS assisted with manuscript preparation and revision.

## Pre-publication history

The pre-publication history for this paper can be accessed here:


